# Recent Advances in Membrane Technologies for Electronic-Grade Hydrogen Peroxide Purification and Concentration

**DOI:** 10.3390/membranes16070229

**Published:** 2026-07-01

**Authors:** Canli Zhang, Jiaofei Lei, Wenpeng Li, Penglin Yang, Wenjia Wu, Feifei Wang, Weizhi Song, Suilu Yue, Guangwei Cheng

**Affiliations:** 1 Intelligent Vehicle Engineering College, Luoyang Institute of Science and Technology, Luoyang 471000, China; znclgcxy@lit.edu.cn (C.Z.); ljff@lit.edu.cn (J.L.); wff240704@163.com (F.W.); wz_song@whut.edu.cn (W.S.); yslxmuhg@163.com (S.Y.); chenggwtom@163.com (G.C.); 2 School of Chemical Engineering, Zhengzhou University, Zhengzhou 450001, China; liwenpeng@zzu.edu.cn

**Keywords:** hydrogen peroxide purification, membrane separation, electronic-grade chemicals, oxidative stability, cascade system

## Abstract

Hydrogen peroxide (H_2_O_2_) is widely used in semiconductor cleaning and etching, where ultralow levels of metallic, anionic, organic, and particulate impurities must be strictly controlled. Industrially produced H_2_O_2_ therefore requires extensive downstream purification before it can meet electronic-grade specifications. Conventional purification routes based on distillation or rectification, adsorption, ion exchange, and final filtration are technically mature, but they remain constrained by substantial energy consumption, multiple treatment stages, chemical regeneration, secondary waste generation, and safety risks associated with H_2_O_2_ decomposition. This review critically evaluates membrane technologies for purifying and concentrating electronic-grade H_2_O_2_. Microfiltration and ultrafiltration are discussed as front-end clarification processes, nanofiltration as an intermediate impurity-load-reduction step, and reverse osmosis as the membrane process with the strongest direct experimental for ionic-impurity removal from concentrated H_2_O_2_. Pervaporation and membrane distillation are assessed as emerging water-removal technologies, although their industrial applicability remains insufficiently validated. Membrane material strategies, including oxidation-resistant polymers, inorganic and hybrid membranes, antioxidant-containing composites, and emerging MOF- and two-dimensional-material-based membranes, are also evaluated. Particular attention is paid to the limited direct evidence available for emerging materials and to the risks of H_2_O_2_ decomposition, material leaching, particle release, and deterioration of membrane selectivity. The available evidence indicates that membrane processes are currently more appropriately regarded as complementary clarification, purification, polishing, or concentration units rather than complete replacements for established industrial technologies. Future studies should prioritize long-term oxidative stability, ppb- and ppt-level impurity validation, low H_2_O_2_ loss, module-material compatibility, process safety, and continuous pilot-scale techno-economic assessment.

## 1. Introduction

**Figure 1:**
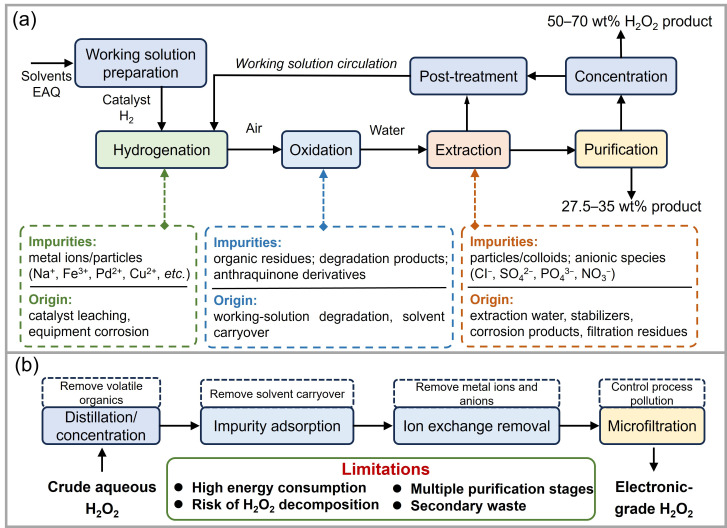
Schematic illustration of the anthraquinone oxidation process for industrial H_2_O_2_ production and representative impurity sources (**a**) and conventional industrial purification route for electronic-grade H_2_O_2_ (**b**).

Hydrogen peroxide (H_2_O_2_) is a versatile chemical with broad applications in chemical synthesis [1], pulp and paper bleaching [2], wastewater treatment [3,4], and, most notably, semiconductor manufacturing [5,6]. In the semiconductor industry, electronic-grade H_2_O_2_ is extensively employed for silicon wafer cleaning and etching, where ultrahigh purity is required to prevent contamination that can compromise device yield [7,8]. Global H_2_O_2_ production exceeds 5.5 million tons annually and is dominated by the anthraquinone autoxidation process [9,10]. The anthraquinone oxidation (AO) process is a mature cyclic liquid-phase route used for large-scale H_2_O_2_ production. However, various impurities may be introduced into the product during hydrogenation, oxidation, extraction, and downstream processing (Figure 1a). Trace metal ions, including Fe^3+^, Na^+^, Pd^2+^, and Cu^2+^, may originate from catalyst carryover or corrosion of reactors and pipelines. Anionic impurities, including phosphates, sulfates, nitrates, chlorides, and other oxygen-containing anions, can be introduced from stabilizers, pH regulators, extraction water, or auxiliary chemicals. In addition, residual organic impurities such as anthraquinone derivatives, solvent residues, and degradation products of the working solution may remain after phase separation and extraction. Particulate impurities and colloids may also originate from catalyst residues, filter aids, corrosion products, or suspended degradation products. These impurities prevent industrial-grade H_2_O_2_ from satisfying the stringent requirements of electronic-grade applications. Additional downstream is therefore required after conventional production and concentration [11,12]. For instance, industrial-grade 30–35 wt% H_2_O_2_ may contain metal ion impurities at parts-per-million (ppm) levels [13], whereas electronic-grade H_2_O_2_ requires these contaminants to be reduced to parts-per-billion (ppb) or even parts-per-trillion (ppt) levels [14,15]. The Semiconductor Equipment and Materials International (SEMI) C30-1110 standard classifies H_2_O_2_ into five quality grades, as summarized in Table 1 [16]. Among them, the most stringent Grade 5 specification requires total metal cation concentrations below 10 ppt in a 30 wt% H_2_O_2_ solution. This purity requirement is far more stringent than that of conventional industrial-grade H_2_O_2_. Post-production purification to ultrahigh-purity levels is therefore indispensable.

**Table 1:** SEMI C30-1110 electronic-grade H_2_O_2_ specifications.

Grade (30% H_2_O_2_)	Max Anionic Impurity	Max Cationic Impurity
Grade 1	<2 ppm	<10 ppb
Grade 2	<0.2 ppm (200 ppb)	<5 ppb
Grade 3	<0.2 ppm (200 ppb)	<1 ppb
Grade 4	<30 ppb	<100 ppt
Grade 5	<30 ppb	<10 ppt

Electronic-grade H_2_O_2_ is commonly produced through a multistep process involving vacuum distillation or rectification, adsorption, ion exchange, and final filtration (Figure 1b) [17,18,19]. In a representative process, crude H_2_O_2_ solution is first subjected to vacuum distillation or rectification for bulk concentration and the removal of volatile or organic impurities. Vacuum operation lowers the boiling temperature and reduces thermal stress on H_2_O_2_, making it suitable for bulk concentration and volatile-impurity removal. The partially purified H_2_O_2_ is then treated by advanced ion-exchange processes, usually involving successive cation- and anion-exchange steps, to remove trace metallic and anionic impurities. Depending on product specifications, additional microfiltration steps may also be included to obtain high-purity or electronic-grade H_2_O_2_ [20,21]. Although such integrated processes can deliver high-purity products, they still have several limitations. Vacuum distillation/rectification is suitable for bulk concentration and volatile-impurity removal, but highly purified H_2_O_2_ production requires strict control of temperature, residence time, construction materials, and trace catalytic impurities to avoid decomposition under unfavorable thermal or catalytic conditions [21,22]. Ion-exchange resins can efficiently remove selected ionic species, with reported removal efficiencies exceeding 98% [23]; however, simultaneous deep removal of cationic and anionic contaminants usually requires multiple resin beds, careful pretreatment, and additional conversion or washing steps, which may introduce secondary contamination [19]. Resin degradation, particle shedding, possible organic leaching, and acidic or alkaline regeneration waste are also important concerns for ultrapure H_2_O_2_ production [24]. Common adsorbents, such as stannic oxide [25], zirconium phosphate [26], alumina [27], and activated carbon [28], typically exhibit selectivity for a limited range of impurities, and their removal efficiency is often insufficient to meet increasingly stringent electronic-grade specifications. These limitations highlight the need for milder, more efficient, and inherently safer purification technologies that can meet the growing demand for ultrapure H_2_O_2_.

Membrane separation technology has attracted increasing attention as a complementary approach for H_2_O_2_ purification and concentration [29,30,31]. For example, pressure-driven membrane processes such as nanofiltration (NF), ultrafiltration (UF), and reverse osmosis (RO) can be operated without chemical regenerants or precipitants, thereby minimizing secondary contamination and regenerant waste relative to conventional resin-based processes [32,33]. A previous modeling study estimated that an integrated countercurrent RO cascade could generate a profit of USD 76.6 million at a target production capacity of 9000 tons/year of electronic-grade H_2_O_2_ [34]. Furthermore, the modular configuration of these systems facilitates incremental installation or parallel scale-up within existing purification lines. However, these potential benefits must be weighed against inherent membrane-specific challenges, including high operating pressures, membrane oxidative degradation, H_2_O_2_ loss via catalytic decomposition, module-material compatibility, stringent pretreatment requirements, and prohibitive membrane replacement costs. Typical membrane processes include microfiltration (MF), UF, NF, RO, pervaporation (PV), and membrane distillation (MD). However, it should be noted that different modalities target distinct impurity categories and are therefore not directly interchangeable. UF or MF can be implemented as front-end clarification steps to remove catalyst fines, colloids, suspended solids, and resin fragments [35]. Subsequently, NF can reduce multivalent metal ions and larger organic residues, thereby mitigating the impurity and fouling loads on downstream units [36]. RO is highly effective for deep removal of dissolved ionic species [37], including trace metal cations and anionic pollutants, owing to its dense selective layer that operates via solution–diffusion and electrostatic-exclusion mechanisms, while allowing water and H_2_O_2_ to permeate. In contrast, PV and MD mainly contribute to H_2_O_2_ enrichment via preferential water removal through vapor-phase or sorption–diffusion transport [38,39]. Therefore, a single membrane process is unlikely to simultaneously meet both the purity and concentration requirements mandated for electronic-grade H_2_O_2_ production.

Accordingly, membrane technology should be conceptualized primarily as a complementary unit in hybrid purification systems rather than as a mere substitute for conventional vacuum distillation/rectification or ion exchange. The primary impetus for integrating membrane separation is not to replace these mature industrial operations, but to explore whether membrane units can mitigate impurity loads, diminish downstream resin consumption, enhance process flexibility, or afford milder polishing stages within existing purification trains. A viable industrial framework could combine the high-throughput concentration capability of vacuum distillation/rectification with the selective impurity-removal capability of membrane processes. The execution of this strategy hinges upon sufficient membrane oxidative stability, module compatibility, minimal H_2_O_2_ loss, and long-term operational safety. The application use of membrane technology for H_2_O_2_ purification dates back to patents from the 1980s [40]. However, concerns regarding the chemical compatibility of membrane materials with this strong oxidant profoundly restricted the volume of peer-reviewed studies for several decades. Over the past 15 years, intensifying research efforts in H_2_O_2_ membrane purification have substantially deepened the understanding of process feasibility, attainable purity, and long-term membrane durability [41,42]. Regrettably, the preponderance of published literature remains confined to short-term laboratory tests, simplified feed solutions, or individual membrane processes. Critical issues such as long-term oxidative aging, module-scale safety, impurity accumulation, and compatibility with industrial H_2_O_2_ production lines have not yet been systematically evaluated.

**Figure 2:**
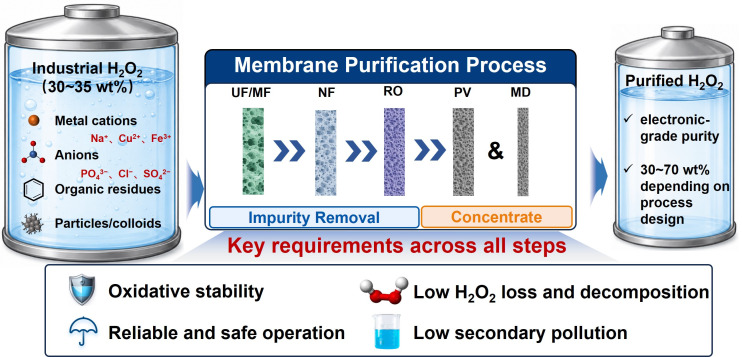
Schematic overview of membrane-based strategies for hydrogen peroxide purification and concentration.

This review systematically examines recent progress in membrane-based H_2_O_2_ purification and concentration processes. As illustrated in Figure 2, pressure-driven membrane processes, including RO, NF, and UF/MF, are discussed with an emphasis on their roles in removing trace ions, multivalent contaminants, particles, and colloids. Furthermore, phase-change or vapor-transport membrane processes, such as PV and MD, are evaluated for their potential in water removal and H_2_O_2_ enrichment. Recent innovations in membrane materials and modification strategies are also explored, encompassing oxidation-resistant polymeric membranes, inorganic membranes, metal–organic framework (MOF)-based hybrid membranes, and two-dimensional (2D) nanomaterial membranes. Moreover, key challenges associated with industrial implementation are critically addressed, including membrane lifetime, the mitigation of oxidative degradation, process integration, and underlying economic and safety considerations. Unlike previous reviews that primarily focus on H_2_O_2_ production or general membrane separation, this review specifically evaluates membrane-based purification and concentration strategies for electronic-grade H_2_O_2_, with a dedicated emphasis on oxidative stability, impurity control, and industrial scalability. This article is a narrative review informed by a structured literature search. Bibliographic databases (e.g., Web of Science and Scopus) and Google Scholar were systematically searched, together with relevant publisher platforms. Patent databases were queried separately. The final literature search was concluded on 9 April 2026. The search covered publications from approximately 1980 to 2026, with particular emphasis on the past 15 years. The main search terms included “hydrogen peroxide purification”, “electronic-grade hydrogen peroxide”, “reverse osmosis”, “nanofiltration”, “ultrafiltration”, “pervaporation”, “membrane distillation”, and “oxidation-resistant membranes”. Articles were selected based on relevance to membrane-based separation processes for hydrogen peroxide purification, methodological quality, and publication recency. Duplicates, irrelevant studies, and articles without accessible full texts were excluded. Peer-reviewed studies were utilized as the primary basis for assessing membrane mechanisms, performance, and stability, whereas patents were treated as supplementary evidence for industrial integration and process design. In total, approximately 180 peer-reviewed publications, 5 academic books and 15 patent documents were considered during manuscript preparation, of which 105 peer-reviewed articles, 3 academic books and 7 patent documents were finally included or discussed in the review.

## 2. Membrane Separation Processes for H_2_O_2_ Purification

**Figure 3:**
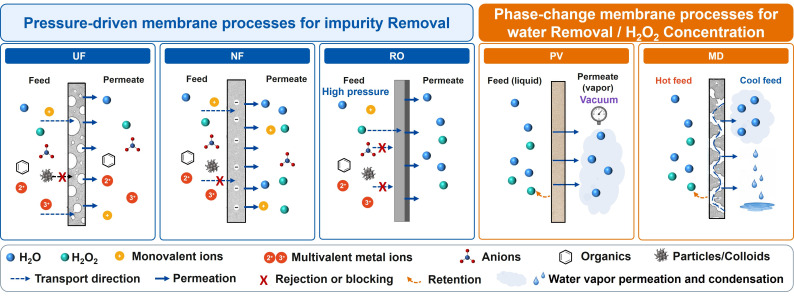
Illustration of various membrane separation processes relevant to H_2_O_2_ purification.

Membrane separation processes for H_2_O_2_ purification and concentration can be better understood when evaluated according to their functional sequence rather than as isolated technologies [38]. As illustrated in Figure 3, UF and MF feature relatively large pores and can therefore serve as front-end clarification steps to remove suspended particles, catalyst fines, colloids, resin fragments, and other particulate impurities from crude H_2_O_2_ streams [35]. NF may then be employed as an intermediate impurity-load-reduction process because its relatively loose selective layer can reject multivalent metal ions, charged organic residues, and larger solutes, while allowing most water and H_2_O_2_ to permeate [36]. RO, characterized by a dense selective layer and sub-nanometer free-volume elements, provides a tighter separation barrier and is therefore more suitable for the rigorous removal of trace dissolved ionic impurities, including metal cations and anionic species, while largely preserving the H_2_O_2_ concentration in the permeate [37]. Following rigorous impurity removal, PV and MD can be implemented for water removal and H_2_O_2_ enrichment. These two processes differ from pressure-driven membranes in that separation is driven by sorption–diffusion coupled with vaporization or by a vapor-pressure gradient across a hydrophobic porous membrane [38,39]. Therefore, the sequence UF/MF → NF → RO → PV/MD reflects a staged purification and concentration paradigm, in which each membrane process addresses a distinct separation target. The representative pore-size range, driving force, operating pressure, separation capacity, suitable process position, and available quantitative performance of these membrane technologies are summarized in Table 2. Because the reported studies differ substantially in feed H_2_O_2_ concentration, impurity species, membrane configuration, operating mode, analytical method, and testing duration, Table 2 is not intended to provide a direct head-to-head performance ranking. Instead, it benchmarks the available information where reported and explicitly distinguishes direct membrane-performance data from integrated-process results, patent disclosures, modeling studies, oxidative-stability tests, and proof-of-concept demonstrations. For integrated purification processes, the final impurity level or claimed SEMI grade is interpreted as the combined effect of multiple unit operations and is not attributed solely to a single membrane step unless the original source provides single-unit performance data. In this section, we therefore discuss the separation mechanisms of the main membrane processes that have been applied or investigated for H_2_O_2_ purification, while also identifying the evidence limits associated with each process.

As summarized in Table 2, the available evidence indicates that the maturity and validation level of different membrane processes vary considerably. MF and UF are mainly supported as clarification or guard-filtration steps, but they do not provide direct removal of dissolved ionic impurities. NF can reduce multivalent ions, larger charged species, and organic residues. However, convincing ppb- or ppt-level monovalent-ion removal under realistic 30–35 wt% H_2_O_2_ conditions has not yet been demonstrated. Among the reviewed processes, RO currently has the most direct quantitative evidence for metal-ion reduction in concentrated H_2_O_2_. For example, commercial RO membranes have been tested in 35 wt% H_2_O_2_, where Na^+^ and Al^3+^ concentrations were reduced from 20895 and 1067 μg/L to 1565 and 87 μg/L, respectively [43]. However, such performance mainly supports lower electronic-grade purification and does not prove suitability for SEMI Grade 4 or Grade 5. Therefore, the reported RO performance should be interpreted as evidence of effective impurity reduction rather than as validation of the highest electronic-grade specifications. Regarding NF, existing studies mainly demonstrate oxidative stability and the partial rejection of multivalent ions and larger solutes; however, convincing ppb- or ppt-level monovalent-ion removal under realistic 30–35 wt% H_2_O_2_ conditions has not yet been unequivocally demonstrated. In the context of PV, the reported BN–GO membrane achieved a marginal H_2_O_2_ concentration increase merely from 0.25 wt% to 0.72 wt%, which should be regarded as proof-of-concept evidence rather than industrially relevant concentration performance [44]. Similarly, while MD has shown potential for concentrating high-concentration H_2_O_2_ streams, electronic-grade impurity-control data remain distinctly limited. Consequently, the current body of evidence supports a staged membrane-assisted purification concept, while also indicating that only RO currently possesses relatively direct evidence for efficacious ionic impurity polishing within concentrated H_2_O_2_ environments.

**Table 2:** Benchmark comparison of representative membrane processes for H_2_O_2_ purification and concentration.

Membrane Process	Representative pore Size/Operating Pressure or Driving Force	Representative Membrane	Feed H_2_O_2_ Concentration/Feed Type	Target Impurity	Removal Efficiency or Concentration Factor	Membrane Stability and H_2_O_2_ Loss	Demonstrated SEMI Grade	Suitable role in an Integrated H_2_O_2_ Purification Train	Ref.
MF	~0.1–10 μm/<2 bar	PVDF, PTFE, or other fluoropolymer microporous filters;	Industrial, crude, or pretreated H_2_O_2_ streams; commonly 30-35 wt% H_2_O_2_	Suspended particles, catalyst fines, resin fragments, colloids, dust particles, and filtration residues	N.R.	N.R.	Not demonstrated for MF alone	Clarification before NF, RO, ion exchange, or final ultrapure filtration	[18,35,39,45]
UF	~0.01–0.1 μm/1–5 bar	Polymeric UF membranes	Crude or pretreated H_2_O_2_ streams; exact feed concentration often not specified ed ions	Colloids, macromolecules, resin fragments, fine particles, and suspended degradation products	N.R.	N.R.	Not demonstrated	Pretreatment or guard membrane to protect downstream NF/RO membranes from particle fouling	[35,39,46,47,48,49,50]
		UF combined with macroligands or chelating agents	Commonly around 30-35 wt%		Approximately 90.2% for Fe^2+^, 89.5% for Al^3+^, and 99.5% for Sn^2+^ in representative examples	H_2_O_2_ loss is N.R.; added ligands may introduce secondary contamination and require downstream removal	Not demonstrated as a complete SEMI Grade 4/5 process		[51]
		SiO_2_-ZrO_2_ ceramic membranes and BTESE-derived organosilica membranes	0.3 wt% and 30 wt% H_2_O_2_ exposure		High model-solute rejection retained after oxidative exposure; direct trace impurity removal in electronic-grade H_2_O_2_ was not demonstrated	Stable after exposure to 0.3wt% H_2_O_2_ for 450 h and 30wt% H_2_O_2_ for 120 h	Not demonstrated		[52]
NF	~0.5–2 nm/5–20 bar	Commercial PES-based NF membranes	1 wt% and 5 wt% H_2_O_2_ exposure	Multivalent ions, larger charged species, organic residues, and oxidative-stability evaluation	N.R. for ppb/ppt-level monovalent ion removal in concentrated H_2_O_2_	Exposure to 5wt% H_2_O_2_ for 5 days	Not demonstrated	Intermediate impurity-load-reduction step before RO or ion exchange	[36,53,54,55,56,57,58,59,60]
		modified PES/TiO_2_ NF membranes				Exposure to 5wt% H_2_O_2_ for 10 days	Not demonstrated		[56]
RO	<1 nm/20–60 bar	Commercial PA and CA RO membranes	35 wt% H_2_O_2_	Dissolved ionic impurities, especially metal cations such as Na^+^ and Al^3+^	Approximately 92.5% removal for Na^+^ and 91.8% removal for Al^3+^	Approximately 64 h	SEMI Grade 2/3, not demonstrated for SEMI Grade 4/5	Final or near-final ionic polishing step, often combined with ion exchange or ultrapure filtration	[40,43,61,62,63,64,65,66]
		Two-stage or multistage RO networks	Modeled H_2_O_2_ purification systems		Model-predicted improvement; no direct experimental removal value	70 h, require experimental verification	SEMI Grade 4/5, require experimental verification		[34,67]
PV	no continuous pores/ vacuum, moderate temperature	BN-GO hybrid pervaporation membrane	Dilute H_2_O_2_ feed, approximately 0.25 wt%	Water removal for H_2_O_2_ enrichment	Approximately 2.9-fold H_2_O_2_ enrichment, from 0.25 wt% to 0.72 wt%	168 h in dilute H_2_O_2_; H_2_O_2_ loss, swelling resistance, and performance in industrial 30-70 wt% H_2_O_2_ remain unverified	N.A.; impurity removal and SEMI grade not demonstrated	Potential mild concentration step after impurity removal	[44,68,69,70,71]
MD	~0.1–1 μm/ near-atmospheric hydraulic pressure	Hydrophobic microporous membrane	69.6 wt% H_2_O_2_	Water vapor removal for H_2_O_2_ concentration	Concentration from 69.6% to 85.4%; yield approximately 80%	Requires strict control of membrane wetting, thermal conditions, material compatibility	N.A.; impurity removal and SEMI grade not demonstrated	Alternative or complementary concentration step for high-concentration H_2_O_2_, requiring strict wetting and safety control	[52,68,72,73,74,75,76]

N.R., not reported; N.A., not applicable.

### 2.1. Ultrafiltration

UF membranes possess relatively large pores of approximately 0.01–0.1 µm and primarily reject colloids, particulate matter, and high-molecular-weight species [46,47]; however, they allow dissolved salts and low-molecular-weight species to pass through almost unhindered [48,49,77]. Consequently, UF is not suitable as a primary purification technology for producing electronic-grade H_2_O_2_, which necessitates the rigorous removal of dissolved ionic impurities is required. Nevertheless, UF can fulfill an important auxiliary role within the H_2_O_2_ purification system. In the industrial anthraquinone process, the crude product may contain fine particles, filtration aids, catalyst residues, or degradation products. UF, or even microfiltration, can be introduced as a front-end clarification step to remove these suspended impurities prior to higher-selectivity membrane processes [50], thereby protecting downstream NF or RO membranes from particle contamination and improving overall operational stability.

Another potential application of UF in H_2_O_2_ purification involves its integration with chelating agents [78]. In this approach, chelating agents bind trace metal ions to form larger complexes, which can then be rejected more effectively by UF [79]. For instance, Pb^2+^ or Cu^2+^ could, in principle, be converted into larger complexed species that are more readily rejected by UF [80]. Although chelation-assisted UF may improve apparent metal rejection, this strategy is difficult to reconcile with ultrapure H_2_O_2_ production, as any added ligand introduces a new contamination source and necessitates additional downstream removal. Consequently, for electronic-grade applications, additive-free purification routes are highly preferred.

In summary, UF mainly serves as an auxiliary or pretreatment step in H_2_O_2_ purification rather than as a final polishing technology. Its principal function is to remove suspended solids, colloids, and other particulate contaminants, thereby protecting downstream NF or RO membranes from fouling. Owing to its low operating pressure and compatibility with high-surface-area hollow-fiber configurations, UF can be conveniently implemented as a front-end barrier within integrated membrane processes. However, to meet stringent final specifications for dissolved ionic contaminants, UF must invariably be followed by a tighter membrane process.

### 2.2. Nanofiltration

NF membranes typically feature pore sizes on the order of approximately 1 nm, slightly larger than those of RO membranes, and generally exhibit charge-based selectivity [46]. NF is therefore often regarded as an intermediate process between UF and RO, or as a “loose RO” process. These membranes retain multivalent ions and larger organic molecules while allowing most monovalent ions and solvent molecules to permeate [53], making them attractive for partial desalting or pretreatment applications. However, in the context of H_2_O_2_ purification, NF is generally insufficient to reduce all ionic impurities to ppt levels, particularly monovalent ions such as Na^+^. Nevertheless, it can serve as an effective pre-purification step or be integrated with RO in a hybrid process [54]. For example, NF could remove multivalent metal contaminants (e.g., Pb^2+^, Fe^3+^, and other transition metals) and larger organic residues from an H_2_O_2_ solution, thereby reducing the impurity burden and fouling potential of a downstream RO unit [55]. Since most water and H_2_O_2_ can permeate through NF membranes, the retained impurities are concentrated in a smaller retentate stream, which can subsequently be further treated, recycled, or managed as waste. Therefore, NF should be regarded primarily as a load-reduction and pretreatment process rather than a final purification step. Its relatively loose selective layer and limited monovalent-ion rejection make it unsuitable as a stand-alone technology for producing high-grade electronic H_2_O_2_. Studies on NF-based H_2_O_2_ purification have provided experimental support for these concepts. For example, the study by Tsehaye et al. showed that commercial PES-based NF (PES 10 and NP030) still exhibited good filtration characteristics after soaking in 1 wt% H_2_O_2_ for 20 days [56].

NF has also been investigated for treating H_2_O_2_-containing waste streams, such as spent semiconductor cleaning baths, with the aim of recovering both H_2_O_2_ and water [57]. In such cases, NF membranes can retain heavy metal ions and organic contaminants in the concentrate stream while allowing H_2_O_2_ and water to permeate, thereby enabling the partial recovery and reuse of the oxidant. Optimized NF membranes have demonstrated the effective removal of multivalent metal ions from dilute H_2_O_2_-containing waste streams, highlighting the value of NF for bulk impurity reduction and resource recovery [81]. However, NF has intrinsic limitations for producing electronic-grade H_2_O_2_. Given that conventional NF membranes generally exhibit limited rejection of monovalent ions, such as Na^+^, as well as very small neutral molecules, single-stage NF is unlikely to meet the most stringent electronic-grade specifications by itself [30,58]. Therefore, NF is more appropriately positioned as a pretreatment or intermediate purification step prior to RO. By removing a portion of divalent ions and larger particles, NF reduces the impurity load and fouling potential of downstream RO membranes, thereby improving the robustness and efficiency of the overall membrane process.

The application of NF in H_2_O_2_ purification must also account for the highly oxidative nature of H_2_O_2_. Common NF membrane materials, including aromatic polyamides and PES, may undergo oxidative degradation during prolonged exposure to H_2_O_2_ [59]. Research by Tsehaye et al. indicates that unmodified commercial NF membranes exhibited rapid performance deterioration after immersion in a 5 wt% H_2_O_2_ solution, whereas a modified PES/TiO_2_ membrane remained stable for more than 20 days [56]. These findings indicate that membrane material design and modification are essential for improving NF durability in oxidative environments, and they will be further discussed in the section on material improvements. Nevertheless, the use of catalytically active inorganic additives requires careful assessment, because improved membrane stability may be accompanied by partial H_2_O_2_ decomposition. In summary, while NF is a valuable component in the H_2_O_2_ purification process and can be used for partial purification, it alone is generally insufficient for reducing monovalent ions to the ppb or ppt level required for high-grade electronic applications [60]. Owing to its relatively high flux and low operating pressure, NF is best suited for integration into hybrid membrane processes, especially as an auxiliary or pretreatment step before RO, where it can help balance system load, mitigate fouling, and enhance overall process efficiency. To date, modified NF membranes have not yet provided convincing evidence for deep monovalent-ion removal under realistic H_2_O_2_ purification conditions. Reported NF modifications mainly improve oxidative stability or multivalent-ion rejection, but they have not clearly demonstrated the reduction of Na^+^, Cl^−^, NO_3_^−^, or other monovalent ionic impurities to ppb- or ppt- levels in concentrated 30–35 wt% H_2_O_2_ streams. Therefore, NF should fundamentally be regarded as a pretreatment or load-reduction unit rather than a final polishing process for electronic-grade H_2_O_2_.

### 2.3. Reverse Osmosis

RO represents one of the most pivotal membrane processes for the ultrapurification of H_2_O_2_ owing to its capacity to reject dissolved ionic impurities while allowing water and H_2_O_2_ to permeate through the membrane [61,62]. Unlike porous membranes, RO membranes rely predominantly on a dense selective layer, wherein separation is governed by solution–diffusion, electrostatic exclusion, and ion hydration effects rather than simple size exclusion [82,83]. In H_2_O_2_ purification, this mechanism is particularly critical because H_2_O_2_ exhibits a molecular size and transport behavior comparable to those of water, whereas hydrated metal ions and anionic species are much more robustly rejected [43]. Therefore, RO can in principle mitigate trace cationic and anionic impurities without substantially inducing the dilution or concentration of H_2_O_2_ product. In addition, RO does not require the addition of chemical regenerants or precipitants, thereby minimizing the risk of secondary contamination. In principle, the impurity-rich concentrate stream can also be recycled or redirected to applications with lower purity requirements, thereby facilitating the minimization of waste emissions. Early experimental studies have validated the effectiveness of RO in eliminating metallic contaminants from H_2_O_2_ solutions. For a systematic overview, the documented RO studies and processes can be classified into three distinct categories.

The first category encompasses commercial RO membrane screening studies, wherein polyamide, cellulose acetate, or related dense composite membranes are evaluated for H_2_O_2_ transmission and metal-ion rejection. For instance, Abejón et al. investigated the impurity separation characteristics of six different commercial RO membranes in a 35 wt% H_2_O_2_ solution and identified that the BE membrane manufactured by Woongjin Chemical was the most suitable candidate for H_2_O_2_ ultrapurification [43]. Specifically, the concentrations of ${\mathrm{Na}}^{+}$ of Na^+^ and Al^3+^ concentrations decreased from 20895 and 1067 μg/L to 1565 and 87 μg/L, respectively. These studies provide direct experimental evidence supporting the feasibility of RO-based H_2_O_2_ ultrapurification.

**Figure 4:**
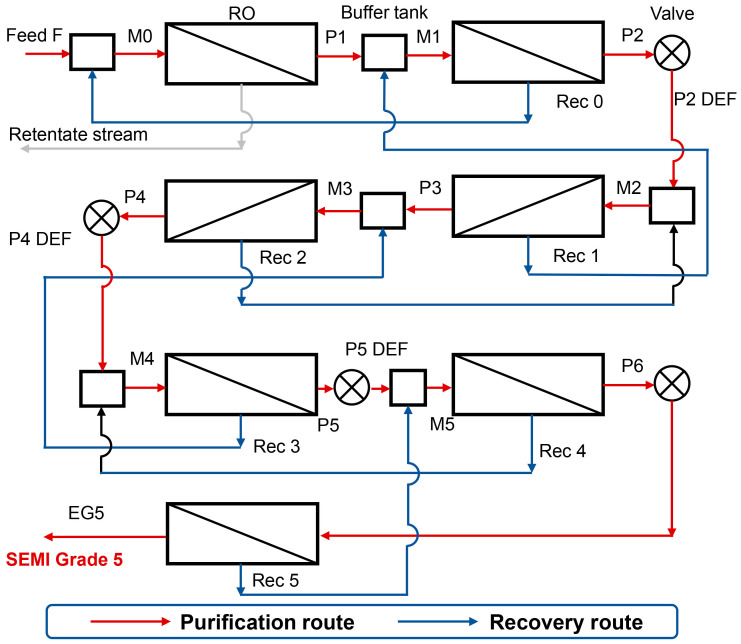
Schematic diagram of cascade RO system for electronic-grade H_2_O_2_ purification Copyright©2012, the John Wiley and Sons [34].

The second category comprises multistage or cascade RO process designs, which employ mathematical modeling and system optimization to explore how single-stage limitations can be overcome via advanced process configuration. Although RO is capable of purifying H_2_O_2_, the reported performance was primarily associated with lower semiconductor-grade specifications, and the ability of a single RO stage to achieve the ppt-level impurity limits required for the highest electronic grades has yet to be demonstrated. To address this gap, several studies developed mathematical models and process-optimization strategies for multistage RO purification. Countercurrent cascade configurations were proposed to improve impurity removal while maintaining high H_2_O_2_ recovery. Modeling results suggested that relatively low electronic-grade specifications may be attainable using a limited number of RO stages, whereas achieving the highest purity grade may require up to seven RO stages, in tandem with larger membrane areas and more complex cycling configurations (Figure 4) [67]. Other studies optimized RO networks containing membrane modules, mixers, splitters, and recycle streams to minimize membrane area or operating costs and to enable the simultaneous production of H_2_O_2_ streams with different purity levels [34]. While these studies are valuable for process design, they should be interpreted as computational assessments based on experimentally fitted transport parameters rather than as direct demonstrations of semiconductor-grade product quality.

The third category encompasses research on membrane lifetime and oxidation stability. The application of RO in H_2_O_2_ purification is strongly constrained by the oxidative stability of polymeric membranes, particularly polyamide selective layers [84]. Ling et al. reported that the oxidative degradation of polyamide selective layers occurs rapidly under conditions of high H_2_O_2_ concentrations, prolonged exposure, elevated temperatures, or the presence of transition metal impurities that promote free radical formation [63]. For prolonged RO operation in percent-level H_2_O_2_ solutions, protective measures or more oxidation-resistant membrane materials are imperative. Strategies such as surface coating, antioxidant incorporation, selective layer modification, and cascade operation optimization may help prolong the lifespan of the membrane [64]. Furthermore, Lin et al. utilized a GO-modified polyamide selective layer, which demonstrated improved oxidative stability [85].

The key performance characteristics of representative RO systems for H_2_O_2_ purification are summarized in Table 3. Because detailed peer-reviewed studies directly targeting semiconductor-grade H_2_O_2_ remain limited, patent-disclosed industrial processes are also discussed as supplementary evidence for practical process integration, whereas general RO theory or modeling references are used exclusively to support mechanistic interpretation. Overall, RO is most effective as a deep ion removal unit, especially for the elimination of metal cations and anionic contaminants from pretreated H_2_O_2_ streams. However, its industrial application still depends on resolving several technical challenges, including the long-term oxidative stability of the selective layer, reliable operation in percent-level H_2_O_2_, the control of metal release from modules and auxiliary components, validation at ppt-level impurity specifications, and integration with final UF or other polishing units.

### 2.4. Pervaporation (PV) and Membrane Distillation (MD)

RO, NF, and UF are primarily employed for impurity removal and generally do not substantially alter the H_2_O_2_ concentration. In contrast, membrane processes such as PV and MD can concentrate H_2_O_2_ by selectively removing water from the solution. Conventionally, H_2_O_2_ has been concentrated from approximately 30 to 70 wt% via distillation. However, this thermal process is energy-intensive and requires stringent safety control [89]. In this context, PV and MD have attracted increasing interest as membrane-based concentration technologies, as they enable water/H_2_O_2_ separation under relatively mild operating conditions [68,72].

**Figure 5:**
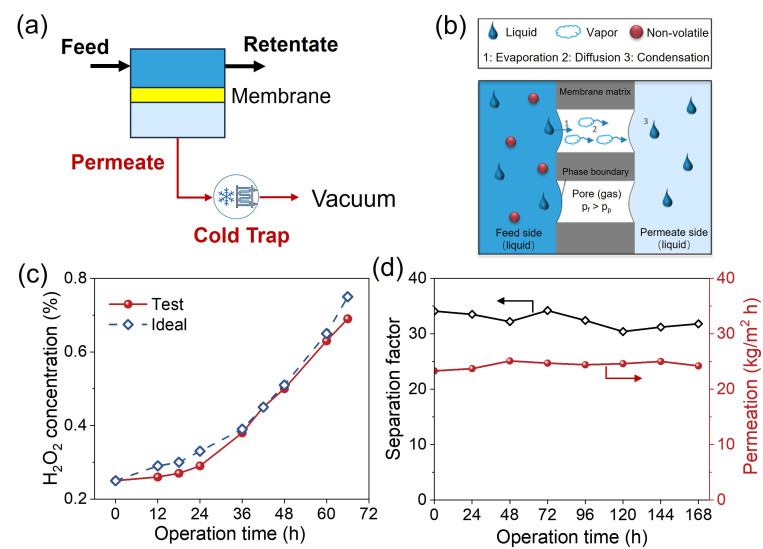
Membrane-based concentration mechanism of H_2_O_2_ through PV (**a**) and MD (**b**) [94] Copyright©2016, the American Chemical Society. Concentration testing (**c**) and long-term stability (**d**) of BN-GO hybrid membrane Copyright©2022, Elsevier [44].

PV is a membrane separation process in which a liquid feed is brought into contact with one side of a dense selective membrane, while the permeate side is maintained under vacuum or swept with an inert gas to continuously remove the permeating vapor (Figure 5a) [90,91]. Separation is governed by the preferential sorption and diffusion of specific components through the membrane, followed by evaporation on the permeate side. PV has been investigated as a means to enrich H_2_O_2_ by preferentially allowing water to permeate and evaporate through the membrane [92,93]. Operation at moderate temperatures (often 40–60 °C), combined with reduced pressure on the permeate side, can promote water removal while mitigating the thermal decomposition of H_2_O_2_.

Recently, graphene oxide (GO)-based membranes have shown promising performance for the PV concentration of H_2_O_2_ [62]. GO laminates contain 2D nanochannels that can facilitate rapid water transport while restricting H_2_O_2_ permeation, thereby enabling effective H_2_O/H_2_O_2_ separation [69]. Wang et al. constructed a hybrid PV membrane based on biomimetic principles by co-assembling rigid hexagonal boron nitride (BN) nanosheets with flexible GO nanosheets [44]. The resulting BN–GO membrane exhibited high water selectivity and robust stability in H_2_O_2_ solutions. In a 72 h test, the membrane concentrated a 0.25 wt% H_2_O_2_ solution to 0.72 wt%, corresponding to a nearly threefold enrichment, as shown in Figure 5c. It achieved a water/H_2_O_2_ separation factor of approximately 35 and a permeation flux of 24.2 kg/m^2^·h (Figure 5d). The incorporation of BN nanosheets helped suppress GO swelling and improved oxidative stability, allowing the membrane to maintain a separation factor above 30 during a week-long operation. These results provide proof-of-concept evidence for water-selective PV. However, PV should currently be regarded as an early-stage proof-of-concept strategy for H_2_O_2_ concentration rather than a technology close to industrial deployment. Although the BN–GO membrane demonstrates selective water removal and provides useful mechanistic insight, the reported concentration increase from 0.25 wt% to 0.72 wt% remains far below the 30–70 wt% range relevant to industrial H_2_O_2_ production and purification. Future PV studies must verify performance using higher-concentration H_2_O_2_ feeds, evaluate H_2_O_2_ loss and decomposition, and demonstrate long-term membrane stability, swelling resistance, module compatibility, and safe operation before practical relevance can be established.

MD is another membrane-based concentration technique wherein a hydrophobic microporous membrane separates a heated liquid feed from a cooler permeate side. Unlike conventional distillation, MD can remove water from H_2_O_2_ solutions at temperatures well below the normal boiling point, since the driving force is the vapor pressure gradient rather than bulk boiling (Figure 5b) [71,95]. This feature makes MD particularly attractive for gently concentrating H_2_O_2_ under near-ambient pressure while mitigating thermal stress and the risk of peroxide decomposition. A representative example was reported by Parrish et al., who proposed an MD-based method capable of concentrating H_2_O_2_ from 69.6% to 85.4% with a yield of approximately 80% [76]. In principle, MD is less constrained by osmotic pressure than pressure-driven membrane processes and can therefore concentrate solutions to high solute levels [73]. However, MD remains at the laboratory or pilot scale, primarily limited by the requirement that all component materials (membranes and modules) must exhibit sufficient tolerance to H_2_O_2_. Moreover, preventing membrane pore wetting by H_2_O_2_ is crucial, because any liquid breakthrough would compromise the vapor barrier and separation performance. Nevertheless, laboratory-scale studies have demonstrated the feasibility of MD for H_2_O_2_ concentration when appropriate membrane materials and temperature-control strategies are employed.

Overall, PV and MD provide promising but still immature alternatives to conventional distillation for producing concentrated H_2_O_2_ under milder conditions. By selectively removing water at moderate temperatures (often <60 °C), these processes can substantially reduce thermal stress, energy demand, and safety risks associated with H_2_O_2_ decomposition.

As discussed above, different membrane processes contribute to H_2_O_2_ purification and concentration through distinct separation mechanisms. However, their reported performances cannot be directly compared using a single numerical criterion, because the available studies differ substantially in feed composition, H_2_O_2_ concentration, impurity type, operating pressure, temperature, membrane configuration, and testing duration. More importantly, some reports are based on direct H_2_O_2_ purification experiments, whereas others provide only indirect evidence from oxidative stability tests, waste-stream treatment, patent-oriented studies, or analogous membrane systems. Therefore, Table 4 summarizes these membrane processes in terms of their main process functions, evidence levels, suitable positions in an integrated purification train, major limitations, and current industrial readiness.

## 3. Membrane Materials and Modification Strategies

**Figure 6:**
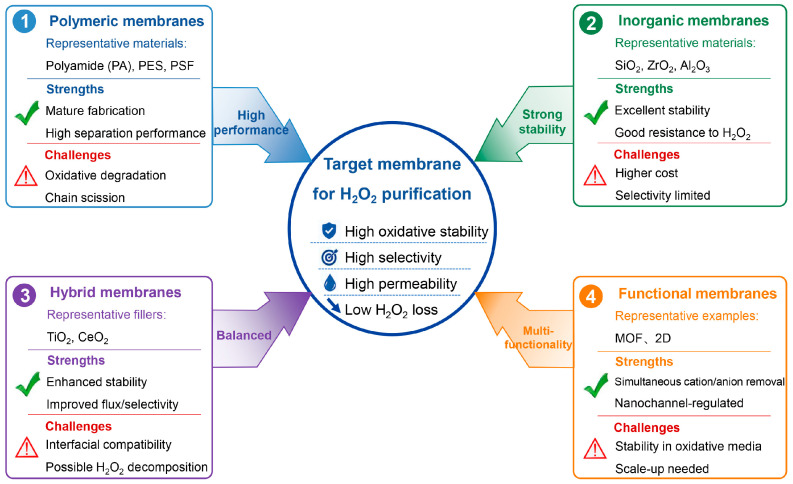
Material-design strategies for oxidation-resistant and multifunctional membranes in H_2_O_2_ purification.

The efficient and stable implementation of membrane-based H_2_O_2_ purification depends not only on the separation process itself but also, critically, on the rational design and optimization of membrane materials. Owing to the strong oxidizing nature of H_2_O_2_, membranes must maintain structural integrity and oxidative stability during long-term operation, while simultaneously providing sufficient selectivity for trace impurity removal and adequate permeation flux. Therefore, the core issue in this field has shifted from whether membranes can be applied to H_2_O_2_ systems to how material design can enable a synergistic balance among stability, selectivity, permeability, and additional functionality. Recent efforts toward this objective have predominantly focused on several material strategies, including intrinsically stable inorganic membranes [74], polymer composite membranes with antioxidant fillers [56], MOF hybrid membranes with multiple adsorption or coordination sites [15], and two-dimensional material membranes capable of regulating nanoscale transport channels [69], as illustrated in Figure 6. A central challenge in membrane material design is that improvements in oxidative stability may not automatically translate into enhanced purification performance. Dense protective layers can reduce flux, catalytic fillers may consume H_2_O_2_, and highly porous structures may compromise selectivity. Therefore, material performance should be evaluated through multiple coupled metrics rather than a single stability indicator. This section discusses these material strategies and analyzes their specific roles in enhancing the feasibility, durability, and separation performance of membrane-based H_2_O_2_ purification.

### 3.1. Polymeric, Inorganic, and Hybrid Membranes: Practical Examples and Applicability

This section commences with a general comparison of polymeric, inorganic, and hybrid membrane systems, as this classification provides the foundational material framework for understanding subsequent membrane modification strategies. Conventional RO/NF membranes are predominantly polymer-based, most commonly aromatic polyamide thin-film composite membranes. However, polymeric materials are inherently vulnerable to oxidative degradation in H_2_O_2_-containing environments. For example, the amide bonds within polyamide selective layers may be attacked by hydroxyl radicals generated during H_2_O_2_ decomposition, leading to chain scission and the subsequent loss of selectivity [96].

In contrast, inorganic membranes, including ceramic membranes based on silica, zirconia, or mixed oxides, generally exhibit substantially higher chemical and thermal stability than conventional polymeric membranes. A notable example was reported by Abejón et al., who evaluated non-commercial SiO_2_–ZrO_2_ ceramic membranes and organosilica membranes derived from bis(triethoxysilyl)ethane (BTES) for UF/NF applications in 30 wt% H_2_O_2_ [52]. The BTES-derived organosilica membrane maintained high solute rejection coefficients, approximately 0.93 for NaCl and 0.97 for glucose, both before and after prolonged H_2_O_2_ exposure, demonstrating excellent oxidative resistance and structural stability in concentrated peroxide media. This example indicates that inorganic or organosilica membranes can be highly advantageous in H_2_O_2_-containing streams where conventional polymeric membranes may suffer from oxidative degradation.

Nevertheless, the practical role of inorganic membranes in electronic-grade H_2_O_2_ production should be defined carefully. Ceramic MF/UF membranes based on silica, zirconia, alumina, or related oxides are more suitable for front-end clarification, where they can remove catalyst fines, colloids, filtration residues, corrosion particles, and suspended degradation products from crude or pretreated H_2_O_2_ streams. However, most inorganic membranes reported to date have not demonstrated the capability to achieve the sub-ppb or ppt-level removal of dissolved ionic contaminants required for high-grade electronic H_2_O_2_. In addition, their relatively broad pore-size distribution, high fabrication costs, brittle mechanical behavior, sealing difficulties, and possible surface-catalyzed H_2_O_2_ decomposition may limit their application as stand-alone final polishing membranes. Therefore, inorganic membranes should be regarded more realistically as oxidation-resistant pretreatment membranes, guard membranes, or mechanically robust support layers in hybrid membrane systems rather than as universal replacements for polymeric RO membranes.

Consequently, increasing attention has been directed toward hybrid membrane systems that combine polymeric matrices with inorganic functional components in order to integrate the advantages of both material classes [75]. Such hybrid strategies aim to simultaneously enhance oxidative stability and separation selectivity. Based on these considerations, current research is progressively shifting from conventional polymeric membranes toward functionalized and hybrid membrane systems specifically designed for the demanding conditions of H_2_O_2_ purification.

### 3.2. Antioxidant Fillers in Polymer Matrices: Stability Enhancement and Contamination Risks

Although functionalized or hybrid systems can improve intrinsic oxidative stability, polymer-based membranes remain dominant in practical pressure-driven separations because of their mature fabrication technologies, scalability, and high separation performance. Consequently, rather than replacing polymers entirely, considerable effort has been devoted to enhancing their resistance to oxidative degradation through the incorporation of functional additives. The main pathway for the chemical degradation of polymer membranes is the breakage of the main chain under strong oxidative conditions or attack by free radicals [97,98]. Integrating multiple components to synergistically enhance antioxidant resistance and mechanical stability is therefore necessary for the design of durable membranes. For example, Tsehaye et al. incorporated TiO_2_-based antioxidant fillers into commercial PES membranes, thereby reducing H_2_O_2_-induced oxidative damage [56]. However, this protection mechanism may rely partly on the catalytic decomposition of H_2_O_2_ into H_2_O and O_2_ at or near the membrane interface, which means that some H_2_O_2_ molecules may be irreversibly consumed during purification. Similarly, inorganic fillers such as CeO_2_, which possess free-radical-scavenging or redox-cycling ability, may enhance membrane oxidative stability [99,100], but may also cause H_2_O_2_ decomposition. Their use therefore requires a delicate balance between improved membrane durability and the preservation of the H_2_O_2_ product. H_2_O_2_ retention and decomposition rates should therefore be considered mandatory performance indicators.

In addition to H_2_O_2_ decomposition, particle contamination is another critical issue for filler-modified membranes. For electronic-grade H_2_O_2_ purification, permeate quality is determined not only by ionic impurity rejection, but also by particle count, metal leaching, total organic carbon (TOC) release, and the absence of membrane-derived contaminants. If inorganic nanoparticles such as TiO_2_, CeO_2_, SiO_2_, or other oxide fillers are not sufficiently immobilized within the polymer matrix, they may detach during long-term operation under cross-flow shear, pressure cycling, oxidative aging, or membrane swelling. Released nanoparticles or filler-derived metal species may then enter the permeate and become unacceptable contaminants for semiconductor applications. Accordingly, antioxidant-filler-modified membranes should not be evaluated solely based on membrane flux, solute rejection, and oxidative stability. For H_2_O_2_ purification, the H_2_O_2_ decomposition rate, oxygen evolution, filler leaching, metal release, TOC contribution, and permeate particle contamination should also be treated as key performance indicators. A membrane that survives longer by catalytically consuming H_2_O_2_ may not be acceptable for industrial purification, because product loss, gas generation, and local safety risks may offset the apparent improvement in membrane durability. Therefore, future studies on TiO_2_-, CeO_2_-, or other oxide-filler-modified membranes should report both membrane aging behavior and H_2_O_2_ loss under realistic H_2_O_2_ concentration, impurity, temperature, and flow conditions. Such risks may be reduced by covalent anchoring, in situ growth, encapsulation beneath a dense selective layer, or post-treatment to remove loosely bound particles, but they require rigorous permeate cleanliness verification before application in ultrapure H_2_O_2_ production.

In contrast, directly improving the tolerance of polymer substrates through chemical modification is more aligned with practical industrial needs. Research has shown that introducing strong electron-withdrawing N-heterocyclic units can enhance polymer oxidative stability by reducing the affinity of the polymer chains for radicals. Based on this, Liu et al. prepared poly(aryl ether ketone) membranes containing N-heterocycles, and test results showed ninefold greater radical tolerance than the unmodified membrane [101]. Nagarajan et al. classified this method of improving polymer oxidation stability through chemical modification into three major groups to facilitate clearer understanding and practical implementation [102]. In the first approach, antioxidant molecules were functionalized with polymerizable groups, which can then be polymerized. The second approach involves the derivatization of a polymerizable monomer with an antioxidant molecule. In the third approach, graft polymerization was used to modify the surface properties of the polymer to tailor it for antioxidant applications. Researchers utilized grafting-to and grafting-from methods to link antioxidants using chemical synthesis or via melt processing.

Surface modification approaches have likewise been investigated to enhance the membrane’s resistance to degradation over time [103,104]. For example, plasma treatment has emerged as a promising technique for the surface modification of membranes, offering a solvent-free approach with potential for industrial-scale application and the ability to selectively alter surface properties without affecting the bulk characteristics of the material [97,105]. A recent study by Heo et al. successfully employed Ar/O_2_ plasma treatment to improve the mechanical and chemical durability of polymer membranes [106]. Meanwhile, Heo et al. emphasized that the Ar/O_2_ plasma dose must be optimized to tailor membrane performance. Overall, both bulk blending and surface functionalization with antioxidant materials have proven effective for improving the long-term stability of polymeric membranes under H_2_O_2_ exposure.

### 3.3. Exploratory Functional Membranes: MOF- and 2D-Nanomaterial-Based Membranes

**Figure 7:**
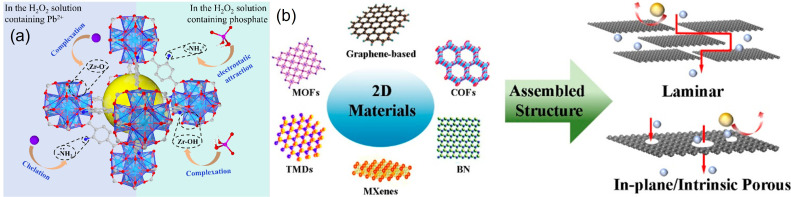
The mechanism of MOF adsorbing both phosphate and Pb^2+^ simultaneously (**a**) [15] Copyright©2024, the American Chemical Society, and (**b**) the regulation of impurity removal characteristics by 2D nanomaterial-based membranes [113] Copyright©2021, the American Chemical Society.

Beyond improving the intrinsic oxidative stability of conventional polymeric membranes, recent material-design studies have explored the introduction of specific adsorption sites and nanoscale transport channels. MOFs and 2D materials represent two conceptually different approaches. MOFs containing Lewis acidic metal centers together with basic or protonatable functional groups may enable concurrent adsorption of metal ions and oxyanions through coordination, electrostatic interaction, or hydrogen bonding (Figure 7a) [107,108]. Two-dimensional layered materials can control the transport of molecules and ions by modulating restricted interlayer channels [109,110]. However, direct experimental evidence supporting their application in concentrated H_2_O_2_ purification remains limited. Therefore, these materials should currently be evaluated as exploratory design strategies rather than established membrane technologies for electronic-grade H_2_O_2_ production.

MOF-containing membranes have attracted attention because their metal nodes, organic linkers, and defect sites can potentially interact with both cationic and anionic impurities. This feature is potentially relevant to H_2_O_2_ purification, wherein the simultaneous removal of trace metal ions and oxyanions remains challenging. For example, defect-rich UiO-66-NH_2_ materials incorporated into polysulfone hollow-fiber membranes have demonstrated simultaneous removal of Pb^2+^ and phosphate from dilute aqueous model solutions [15,31,111]. These results support the general feasibility of adsorption-assisted cation–anion removal. Nevertheless, the experiments were not conducted in concentrated H_2_O_2_, and the reported single-pass removal efficiencies (67.1% for phosphate and 60.1% for Pb^2+^) do not demonstrate compliance with the ppb- or ppt-level impurity limits required for high-grade electronic chemicals.

The incorporation of MOFs may also introduce additional challenges that are less pronounced in conventional pressure-driven membranes. Exposure to H_2_O_2_ may compromise framework integrity, while exposed metal nodes may promote peroxide decomposition or radical generation. Moreover, trace leaching of metal ions, organic linkers, synthesis residues, or partially degraded framework components would be unacceptable in semiconductor-grade H_2_O_2_. Given that the number of adsorption sites is finite, adsorption saturation and regeneration must also be considered. Regeneration chemicals could introduce secondary contaminants and increase process complexity. Consequently, MOF-containing membranes should presently be regarded as adsorption-assisted candidates for pretreatment or polishing applications, rather than direct replacements for RO or ion-exchange processes.

Two-dimensional materials provide an alternative design route by regulating transport through confined nanochannels (Figure 7b). The most relevant example for H_2_O_2_ treatment is a BN–GO hybrid pervaporation membrane, in which rigid boron nitride nanosheets suppress graphene oxide swelling and stabilize the lamellar structure [44]. In a proof-of-concept test, this membrane increased the H_2_O_2_ concentration from approximately 0.25 wt% to 0.72 wt% while maintaining preferential water permeation, indicating that 2D nanochannels can distinguish water and H_2_O_2_ transport under dilute conditions. However, this concentration range is far below the 30–70 wt% H_2_O_2_ levels relevant to industrial and electronic-grade applications, and the result does not demonstrate industrial-scale concentration or trace ionic impurity removal. Moreover, the effectiveness of GO, layered double hydroxides, MoS_2_, and related 2D materials in concentrated H_2_O_2_ remains unverified, and oxidative attack, swelling, delamination, defect formation, interlayer-spacing variation, and nanosheet release may affect long-term performance and product purity [112].

MOF- and 2D-material-based membranes address different aspects of membrane design: MOFs introduce adsorption functionality, whereas 2D materials regulate transport pathways. However, neither approach has yet demonstrated a clear ability to overcome the principal limitations of conventional H_2_O_2_ membrane systems without introducing additional risks. Future studies should evaluate membrane performance under realistic H_2_O_2_ concentrations and impurity levels, with particular attention to H_2_O_2_ recovery and decomposition, impurity rejection, long-term stability, contaminant release, and compatibility with membrane modules and downstream polishing operations.

Beyond membrane separation performance, contamination control is equally critical for electronic-grade H_2_O_2_ production. Potential contamination sources include membrane polymers, inorganic fillers, MOF particles, metal nodes, organic linkers, adhesives, binders, spacers, seals, housings, and piping materials, all of which may release particles, trace metals, organics, or degradation products during long-term exposure to concentrated H_2_O_2_. Therefore, contamination risk should be considered alongside selectivity, permeability, and oxidative stability. Table 5 summarizes the principal contamination sources and corresponding contamination-control considerations relevant to membrane-assisted production of electronic-grade H_2_O_2_. Until long-term pilot-scale validation becomes available, these materials should be regarded as complementary and exploratory candidates rather than mature alternatives to conventional RO, NF, PV, or MD membranes. More importantly, the industrial relevance of these emerging materials will depend not only on their laboratory-scale separation performance, but also on their lifetime, impurity-release behavior, module compatibility, safety, and economic feasibility, as discussed in the following section.

## 4. Towards Industrial Implementation

Membrane-based H_2_O_2_ purification has demonstrated considerable potential at the laboratory scale. However, translating these advances into industrial practice requires careful evaluation of several critical factors, including long-term membrane stability, compatibility with existing production infrastructure, operational safety under strongly oxidative conditions, and overall economic viability. Consequently, this section examines membrane-based H_2_O_2_ purification from four interrelated perspectives: membrane lifetime and replacement, process integration, safety considerations, and techno-economic feasibility. This analysis highlights the major constraints, practical challenges, and future development directions associated with the industrial implementation and scale-up of membrane-based H_2_O_2_ purification.

**Figure 8:**
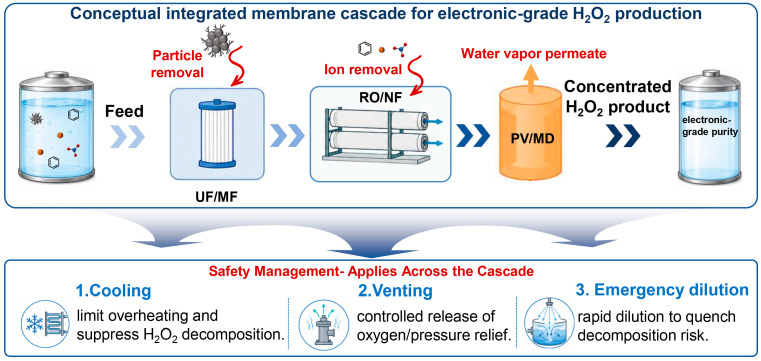
Conceptual cascade membrane system for industrial hydrogen peroxide purification and concentration, integrating front-end clarification, NF/RO-based ionic impurity removal, PV/MD-based water removal, concentrate recycling, and key safety-control measures including cooling, venting, temperature monitoring, automatic shutdown, and emergency dilution.

Leveraging the complementary functions of different membrane processes, a conceptual cascade membrane system for high-concentration H_2_O_2_ is proposed, as illustrated in Figure 8. In this integrated configuration, front-end clarification, utilizing UF or MF, is initially deployed to remove particulate impurities, colloids, and suspended residues that may foul downstream units. Pressure-driven membrane processes, particularly NF and RO, are then employed for staged ionic impurity removal: NF effectively rejects multivalent metal ions and larger contaminants, whereas RO acts as the final high-selectivity polishing step for trace cations and anions. Subsequently, PV or MD is introduced as a mild water-removal unit to increase the H_2_O_2_ concentration while avoiding the high thermal load associated with conventional distillation. The impurity-enriched concentrate generated from the NF/RO stage may be recycled to lower-grade applications or treated separately, thereby reducing waste discharge. Importantly, Figure 8 also emphasizes that industrial membrane implementation must be coupled with process safety controls, including continuous temperature monitoring, rapid cooling, oxygen venting, automatic shutdown, and emergency dilution, because localized H_2_O_2_ decomposition, oxygen accumulation, or pressure excursions could otherwise compromise safe operation. Therefore, the proposed cascade should be regarded not only as a separation flowsheet, but also as an integrated purification–concentration–safety framework for scalable electronic-grade H_2_O_2_ production.

More specifically, the individual elements shown in Figure 8 exhibit varying levels of empirical support. UF/MF clarification is well-established for particle and colloid removal, while RO provides the most direct experimental evidence for mitigating ionic impurities in concentrated H_2_O_2_ streams. NF has been primarily investigated mainly as a pretreatment or impurity-load reduction step, whereas PV and MD have been explored as mild water-removal or H_2_O_2_ concentration methods. Nevertheless, the complete cascade configuration remains conceptual. Continuous integrated operation, impurity-accumulation control, concentrate recycling, safety interlocks, and validated Grade 4/5 production all necessitate pilot-scale demonstration. Therefore, Figure 8 should be interpreted as a proposed integration roadmap rather than as a validated industrial process.

### 4.1. Membrane Lifetime and Replacement

A paramount concern for industry is the operational lifetime of membranes under H_2_O_2_ exposure and the associated replacement frequency. Economic models by Abejón et al. have identified the membrane replacement rate as a key factor governing the competitiveness of membrane-based purification relative to conventional technologies such as ion exchange [65,87]. In particular, the economic advantage of RO can rapidly diminish if membrane degradation necessitates frequent replacement. As previously highlighted, some unmodified polymeric membranes exhibit extremely limited stability in concentrated H_2_O_2_ environments, with effective operating lifetimes ranging from only several days to even a few hours under severe conditions. Abejón et al. analyzed the time-dependent performance of RO membranes in 35 wt% H_2_O_2_ and observed that membrane rejection followed a logistic decay trend, with a pronounced decline occurring after only a few days of continuous operation in the absence of protective strategies [87]. Such short operational lifetimes render large-scale deployment economically impractical. As a practical benchmark, membrane lifetimes of merely hours to several days should be regarded as insufficient for commercial H_2_O_2_ purification. For industrial relevance, membrane modules should ideally operate for at least several months under representative H_2_O_2_ concentration, impurity profiles, temperatures, pressures, and flow conditions. A service life of several months is highly preferable because it aligns better with industrial maintenance cycles and reduces membrane replacement costs. Membrane failure should be defined not only by physical rupture or visible damage, but also by irreversible flux or permeance decline, the loss of impurity rejection, increased H_2_O_2_ decomposition, unacceptable particle or leachate release, excessive pressure drops, or the inability to maintain the target electronic-grade specification. Furthermore, the lack of standardized lifetime testing protocols presents an additional complication. Reported membrane stability may refer to unchanged flux, unchanged rejection, visual integrity, or short-term soaking resistance, which are not equivalent criteria. Future studies should therefore define membrane failure using combined thresholds for flux, selectivity, H_2_O_2_ loss, and impurity release.

### 4.2. Integration into Existing Processes

Once sufficient membrane durability is achieved, the subsequent challenge involves the effective integration of membrane units into practical H_2_O_2_ production and recycling systems. Currently, the production of electronic-grade H_2_O_2_ is dominated by a select number of large chemical manufacturers employing well-established distillation–ion-exchange process schemes [114]. Any new membrane-based process must demonstrate not only high separation performance but also compatibility with existing industrial infrastructure and operational paradigms. One practical implementation strategy involves process retrofitting. As previously noted, RO units could be introduced downstream of conventional distillation columns as polishing steps to partially replace ion-exchange operations or reduce resin loadings. Owing to their modular nature, membrane systems can be readily scaled through parallel module configurations to accommodate industrial throughput requirements. In such hybrid process configurations, RO could serve as a final purification stage to reduce ionic impurities to ultralow levels, thereby decreasing the required size, regeneration frequency, or chemical consumption of ion-exchange units. Similarly, NF may be employed as a pretreatment step to remove multivalent metal contaminants and organic foulants upstream, thereby extending the service lives of both the resin and the RO membrane. Beyond integration into primary production lines, membrane technologies also offer substantial opportunities for H_2_O_2_ recovery and recycling. Specifically, NF could recover an H_2_O_2_-containing permeate from spent semiconductor cleaning solutions while retaining metals and organics within the concentrate. These approaches align with the principles of green manufacturing, waste minimization, and resource recovery, thereby enhancing the economic attractiveness of membrane-based technologies even when implemented as auxiliary recycling units rather than direct replacements for existing purification processes. Ultimately, successful industrial integration will depend on positioning membrane processes as complementary components within existing purification flowsheets rather than as immediate stand-alone replacements for mature industrial technologies. However, the integration of membrane units presents new operational complexities, including pretreatment requirements, pressure control, concentrate management, membrane replacement logistics, and the compatibility of module materials with high-purity chemical handling systems.

### 4.3. Safety Considerations

Safety represents a critical and independent criterion for the industrial deployment of membrane-based H_2_O_2_ purification technologies. Unlike ordinary liquid separation processes, H_2_O_2_ purification involves a strongly oxidative medium whose decomposition may be accelerated by elevated temperatures, catalytic surfaces, metal contaminants, or incompatible construction materials. Therefore, beyond membrane performance and process integration, industrial feasibility must also be evaluated in terms of hazard prevention, materials compatibility, module design, and emergency control strategies. Highly concentrated H_2_O_2_ is a powerful oxidizing agent capable of causing rapid decomposition, oxygen release, fire hazards, or even explosions under unfavorable conditions. This vulnerability is particularly acute for membrane systems because many membrane materials and module components are polymeric and may themselves become susceptible to oxidative degradation or combustion if uncontrolled peroxide decomposition occurs. Consequently, membrane-based purification systems must be carefully designed to maintain operating temperatures, peroxide concentrations, and residence times within safe limits to prevent thermal runaway or localized decomposition events. From an engineering perspective, uncontrolled H_2_O_2_ decomposition constitutes the primary hazard scenario that should be considered during membrane-process scale-up. H_2_O_2_ decomposition is exothermic and generates oxygen, which may lead to localized temperature rises, gas accumulation, oxygen-enriched atmospheres, pressure increases, and self-accelerating decomposition if heat removal or venting is insufficient. Therefore, membrane modules and auxiliary equipment should be equipped with continuous temperature and pressure monitoring, oxygen or gas detection, pressure-relief devices, rupture discs or relief valves, safe vent routing, and automatic shutdown interlocks. Emergency response protocols should include rapid cooling, isolation of the affected module, controlled venting, and emergency dilution with ultrapure water to mitigate H_2_O_2_ concentration and heat-generation risks. Before scale-up, the complete membrane process should undergo a formal process-safety assessment, such as hazard and operability analysis (HAZOP) and, where appropriate, layer of protection analysis (LOPA), to evaluate failure scenarios including membrane rupture, blocked permeate or retentate channels, pump failure, cooling failure, oxygen accumulation, overpressure, sudden H_2_O_2_ decomposition, and containment loss.

Material compatibility constitutes another paramount safety consideration. Compatibility screening should evaluate not only visual degradation or mechanical integrity, but also the H_2_O_2_ decomposition rate, metal leaching, TOC release, particle shedding, swelling, embrittlement, and changes in sealing performance during long-term exposure. Additionally, spacers, seals, adhesives, module housings, and piping components must all exhibit sufficient resistance toward long-term H_2_O_2_ exposure. Crucially, any catalytic additives or functional fillers incorporated into membranes should promote controlled and moderate peroxide decomposition pathways rather than induce rapid radical generation or localized heat accumulation. Rigorous system maintenance and membrane replacement procedures are equally essential. During shutdown, cleaning, or membrane replacement, residual H_2_O_2_ may come into contact with air, metallic surfaces, or incompatible organic materials; therefore, flushing, dilution, ventilation, and decontamination procedures are strictly required to minimize hazard risks.

Among the various membrane processes, MD presents distinct safety challenges because it deliberately operates at elevated temperatures, although still below the boiling point of H_2_O_2_ solutions. For MD systems, module designs incorporating low liquid hold-up volumes, chemically inert materials such as PTFE, temperature monitoring, and pressure-relief mechanisms are particularly critical for preventing uncontrolled peroxide decomposition. More broadly, membrane-based H_2_O_2_ purification systems should be subjected to hazard and operability analyses comparable to those used for conventional distillation units, including the evaluation of worst-case scenarios such as membrane rupture, sudden peroxide decomposition, oxygen accumulation, or pressure excursions. Appropriate mitigation measures, including emergency dilution, rapid cooling, oxygen venting, and automatic shutdown protocols, should therefore be rigorously incorporated into industrial system design.

Despite these challenges, membrane processes also offer several inherent safety advantages. Compared with conventional thermal concentration methods, most membrane separations operate under relatively mild conditions and typically involve lower liquid hold-up volumes, thereby limiting the quantity of H_2_O_2_ exposed to potential decomposition at any given moment. This intrinsic reduction in thermal and reactive inventory can significantly enhance overall process safety provided that membrane systems are appropriately designed and operated.

### 4.4. Cost and Economic Feasibility

A final consideration for industrial implementation is economic feasibility, as the overall cost competitiveness of membrane-based H_2_O_2_ purification is inextricably linked to the issues discussed above. The primary cost drivers for membrane systems encompass membrane module fabrication and replacement, energy consumption by pumping or vacuum systems, and the requisite pretreatment or cleaning chemicals. Several studies indicate that RO-based ultrapurification can be economically competitive for the production of high-purity H_2_O_2_, particularly at stringent purity levels where conventional distillation–ion-exchange processes become increasingly energy-intensive and chemically demanding [86]. In pressure-driven processes such as RO and NF, energy consumption is primarily associated with pumping requirements; conversely, PV and MD may utilize waste heat or other low-grade thermal energy sources, thereby reducing the reliance on the high-grade steam required for conventional distillation. Furthermore, membrane systems present opportunities for low-waste or near-zero-waste operations. For example, impurity-enriched RO concentrate streams could potentially be repurposed for lower-grade H_2_O_2_ production rather than being discarded as waste [115]. Such process integration strategies may improve overall process economics by minimizing material losses and generating recoverable technical-grade H_2_O_2_ streams. For the most stringent electronic-grade specifications, such as SEMI Grade 5, standalone membrane processes alone may still be insufficient, necessitating hybrid combinations with ion exchange or alternative polishing technologies. Nevertheless, membrane processes could substantially diminish the impurity load entering downstream purification units, thereby lowering resin consumption, regeneration frequencies, and overall operational complexity. Despite these promising prospects, membrane durability remains a paramount uncertainty in current economic assessments. Given that membrane replacement contributes substantially to operating costs, even moderate improvements in oxidative stability can profoundly influence process economics. Extending membrane service life from a mere few days to several months could substantially reduce replacement-related costs by an order of magnitude, fundamentally enhancing the commercial viability of membrane-based purification systems. However, current techno-economic evaluations must be regarded as preliminary, as reliable cost data for oxidation-resistant membranes, H_2_O_2_-compatible modules, safety instrumentation, and long-term replacement schedules remain scarce. The economic advantages of membrane systems could be entirely negated if frequent replacement or extensive safety-control infrastructure proves mandatory.

Consequently, the economic competitiveness of membrane-assisted H_2_O_2_ purification should be evaluated within a comprehensive techno-economic framework rather than relying on qualitative comparison. Key capital expenditure (CAPEX) components encompass membrane modules, pressure vessels, pumps, vacuum systems for PV, heat exchangers or temperature-control units, online sensors, pressure-relief systems, emergency dilution units, ultrapure piping, and peroxide-compatible module materials. Correspondingly, critical operating expenditure (OPEX) variables include pumping or vacuum energy requirements, membrane replacement frequencies, pretreatment and cleaning protocols, H_2_O_2_ losses via through permeation or decomposition, concentrate handling, waste-treatment costs, reductions in resin-regeneration, labor, maintenance, and process downtime. These costs should be rigorously benchmarked against conventional vacuum distillation/rectification, adsorption, ion exchange, microfiltration, and final polishing processes. Essential performance indicators should comprise the cost per kilogram of electronic-grade H_2_O_2_, specific energy consumption, H_2_O_2_ recovery rates, membrane lifespans, resin-regeneration frequencies, waste volumes, and product-grade stability. Until comprehensive pilot-scale techno-economic data become available, membrane systems are more accurately characterized as potentially advantageous upgrading or polishing units rather than as proven cost-competitive replacements for conventional distillation–ion-exchange routes.

In summary, the industrial viability of membrane-based H_2_O_2_ purification will not depend on a single performance metric, but rather on the synergistic optimization of membrane durability, process integration, operational safety, and economic performance. For successful industrial implementation, time-dependent pilot-scale operating data are vastly more informative than single-point membrane performance values. Ideally, the feasibility of a membrane-assisted H_2_O_2_ purification process should be evaluated by continuously monitoring normalized membrane permeance or flux, H_2_O_2_ concentration and recovery, representative cationic and anionic impurity levels, TOC, particle counts, pressure drops, temperatures, and gas evolution throughout long-term operation. However, publicly available pilot-plant datasets for membrane-based electronic-grade H_2_O_2_ production are conspicuously scarce. Therefore, rather than presenting unsupported operating curves, Table 6 recommends pilot-scale monitoring parameters and indicative engineering targets for membrane-assisted electronic-grade H_2_O_2_ purification. Because no generally accepted pilot-scale acceptance criteria have yet been established for membrane-based electronic-grade H_2_O_2_ purification, the following targets should be regarded as indicative engineering objectives rather than formal industrial standards. Future pilot tests should demonstrate stable continuous operation, limited flux decline, negligible H_2_O_2_ decomposition, no progressive impurity breakthrough, no detectable membrane-derived leachable, acceptable pressure-drop stability, and sustained product quality under representative H_2_O_2_ concentrations, impurity profiles, temperatures, pressures, and flow conditions. These criteria should ultimately be refined in accordance with the target SEMI grade, specific plant configurations, analytical detection limits, and long-term industrial validation.

## 5. Conclusions and Future Outlook

Membrane-based treatment represents a promising complementary strategy for electronic-grade H_2_O_2_purification. MF/UF, NF, and RO can target distinct impurity classes, whereas PV or MD offers an alternative concentration pathway. However, to date, no integrated membrane configuration has simultaneously demonstrated ultrahigh purity, high H_2_O_2_ recovery, long membrane lifetime, and industrial safety.

A central advance highlighted in this review is the shift from process feasibility toward materials-enabled functionality. Antioxidant-filled polymer membranes, inorganic and organic–inorganic hybrid membranes, MOF-based adsorptive membranes, and two-dimensional lamellar membranes have broadened the range of material concepts under investigation for membrane systems operating in oxidative H_2_O_2_ environments. These material innovations not only improve membrane durability but may also offer additional functionalities, although empirical evidence in concentrated H_2_O_2_ remains limited. In particular, framework-based membranes, including MOF-derived and other emerging porous organic framework systems, offer a promising platform for engineering tailored binding sites and ordered transport channels for ultratrace impurity removal.

Despite these advances, the available evidence does not yet support the conclusion that membrane-based H_2_O_2_ purification is nearing widespread to broad industrial implementation. Many reported studies remain confined to short-term tests, simplified feed solutions, relatively low H_2_O_2_ concentrations, and proof-of-concept membrane configurations. Therefore, membrane technology should currently be regarded as a promising complementary strategy for upgrading existing H_2_O_2_ purification trains rather than as a mature replacement for conventional vacuum distillation/rectification, adsorption, or ion exchange. Its practical value will depend on whether membrane units can be seamlessly integrated into existing industrial processes while maintaining product purity, H_2_O_2_ recovery, long-term stability, safety, and economic feasibility.

Future work should focus on five priorities. (i) Membrane lifetime must be extended from days or short-term laboratory exposure to commercially meaningful operation (several months) under realistic and concentrated H_2_O_2_ conditions. (ii) Material and module compatibility must be verified across all components—including membranes, fillers, supports, adhesives, seals, spacers, housings, and piping, with particular attention devoted to metal leaching, TOC release, particle shedding, and H_2_O_2_-induced degradation. (iii) Membrane processes must demonstrate reliable impurity removal at SEMI Grade 4 or Grade 5 standards, especially achieving ppt-level control of both cationic and anionic contaminants. (iv) H_2_O_2_ loss must be minimized by rigorously monitoring permeation loss, decomposition rate, and oxygen generation. (v) Pilot-scale techno-economic validation is required, incorporating realistic industrial feeds, continuous operation, membrane replacement frequency, energy demand, CAPEX, OPEX, waste-treatment costs, safety controls, and rigorous comparisons with established distillation–ion-exchange purification routes. Addressing these priorities will determine whether membrane-assisted purification can evolve from laboratory feasibility toward practical industrial viability for electronic-grade H_2_O_2_ production.

## Figures and Tables

**Table 3:** RO membrane systems and performance.

RO System/Process type	Membrane or Configuration	Key Performance Characteristics	Main Limitations	Ref.
Early RO process for high-purity H_2_O_2_	Aromatic polyamide, polypiperazineamide, polysulfone, polyacrylonitrile RO membranes	H_2_O_2_ and water permeate; ionic impurities retained in concentrate	Patent-level disclosure; limited detailed membrane-performance data	[40]
Commercial RO membrane screening	Commercial PA and CA RO membranes, e.g., BE membrane from Woongjin Chemical	Metal-ion rejection commonly around or above 90%; Cu^2+^ rejection reported around 96%; boron rejection lower	Mostly lab-scale; mainly SEMI Grade 1, Grade 4/5 not demonstrated. limited long-term data	[43]
Multistage or cascade RO	Two-stage or multistage RO network	Improves impurity rejection beyond single-pass operation; allows process optimization of pressure, area, recovery, and product quality	Modeling/design work; not direct proof of SEMI Grade 4/5 production	[65,86]
RO lifetime and stability studies	Commercial PA and CA RO membranes	Evaluates flux decline, rejection stability, and membrane degradation in oxidizing media	Test conditions milder than real industrial streams	[87]
Recent patent-disclosed integrated route	RO device combined with rectification, ion exchange and ultrapure filtration	RO used as one unit in an integrated electronic-grade H_2_O_2_ production process	Patent disclosure; detailed membrane lifetime and impurity data may be limited	[66]
High-concentration RO purification process	One- or two-membrane RO process for >50 wt% H_2_O_2_	RO used for purification of stabilized high-concentration H_2_O_2_ streams	Not necessarily focused on semiconductor Grade 4/5 specifications	[88]

**Table 4:** Comparative assessment of evidence level, process role, and industrial applicability of membrane technologies for H_2_O_2_ purification and concentration.

Membrane Process	Main Role in H_2_O_2_ Treatment	Evidence Level	Suitable Process Position	Key Limitations	Industrial Readiness
RO	Deep removal of ionic impurities	Relatively strong for ionic removal, but mostly Grade 1-level evidence	Final or near-final polishing step	Polyamide oxidation, limited evidence for Grade 4/5	Medium
NF	Removal of multivalent ions and organic residues	Moderate, mainly pretreatment or waste-stream recovery	Pretreatment before RO	Poor monovalent-ion rejection; insufficient for ppt-level purity	Medium–low
UF/MF	Removal of particles, colloids, catalyst residues	Indirect but technically reasonable	Front-end clarification	Cannot remove dissolved ions	Medium
PV	Water removal for H_2_O_2_ enrichment	Emerging proof-of-concept evidence	Concentration step	Mostly low-concentration tests; scale-up uncertain	Low
MD	Water removal under mild thermal conditions	Limited; often technical reports or patent-oriented evidence	Alternative concentration step	Wetting, H_2_O_2_ loss, safety control, limited peer-reviewed data	Low
Hybrid/cascade systems	Integrated purification and concentration	Conceptual or partially validated	Full-process design	Inter-stage compatibility, safety, cost, lifetime	Low–medium

**Table 5:** Potential contamination sources and contamination-control considerations in membrane-assisted electronic-grade H_2_O_2_ purification.

Component	Potential Contamination Source	Possible Contaminants	Main Concern for Electronic-Grade H_2_O_2_
Polymeric membrane layer	Oxidative degradation	TOC, polymer fragments, degradation products	Increased TOC and organic contamination
Inorganic fillers (TiO_2_, CeO_2_, SiO_2_, etc.)	Particle detachment or dissolution	Nanoparticles, trace metals	Particle contamination and metal release
MOF particles	Framework degradation	Metal ions, organic linkers	Trace metal contamination and TOC increase
MOF metal nodes	Oxidative dissolution	Zr^4+^, Fe^3+^, Cu^2+^, Co^2+^, Al^3+^, etc.	ppt-level metal contamination
Organic linkers	Chemical degradation	Organic fragments, TOC	Semiconductor-grade organic contamination
GO/2D materials	Delamination or nanosheet release	Nanosheets, carbonaceous particles	Particle contamination
Adhesives and binders	Chemical attack by H_2_O_2_	Organic extractables	TOC increase
Feed spacers	Mechanical abrasion and aging	Polymer particles	Particle release
O-rings and seals	Oxidative aging	Organic fragments, additives	Extractables and leachable
Module housings	Corrosion or degradation	Metals, organics	Secondary contamination
Pumps, valves, piping	Corrosion and wear	Fe^3+^, Ni^2+^, Cr^3+^, Cu^2+^, particles	Trace-metal contamination
Instrumentation and sensors	Wetted-material corrosion	Metal ions	Local contamination sources

**Table 6:** Recommended pilot-scale monitoring parameters for membrane-assisted electronic-grade H_2_O_2_ purification.

Parameter to Monitor	Typical Expression	Purpose in Pilot-Scale Validation	Indicative Engineering Target/Acceptance Consideration
Membrane permeance/flux	L/m^2^ h bar or normalized J/J_0_	Evaluates of membrane fouling, compaction, and oxidative degradation	No severe or progressive irreversible decline; the flux decline should remain within a predefined project-specific limit
H_2_O_2_ concentration	wt% in feed, permeate, and retentate	Tracks of H_2_O_2_ transmission, dilution, or concentration behavior	No uncontrolled dilution or concentration drift
H_2_O_2_ recovery/loss	% recovery or decomposition rate	Determines of whether membrane operation consumes or decomposes H_2_O_2_	H_2_O_2_ decomposition or loss should be negligible or below the process-defined economic and safety thresholds
Metal impurities	ICP-MS, ppt–ppb level	Verifies of electronic-grade cation control	Should meet the target SEMI grade or the predefined polishing-stage specification
Anionic impurities	IC, ppb level	Verifies removal of sulfate, phosphate, chloride, nitrate, and other anions	Should meet the target SEMI grade or downstream process requirement
TOC	ppb level	Detects of organic leaching from membrane or module materials	No upward TOC trend attributable to membrane or module leaching; TOC should remain below product specifications
Particle count	Particles/mL at defined size thresholds	Evaluates of particle release and semiconductor-grade cleanliness	No membrane-derived particle release; particle counts should remain within the target electronic-grade specifications
Pressure drop	bar	Indicates of fouling, blockage, or module instability	No continuous increase that would indicate fouling, blockage, gas accumulation, or module instability
Temperature	°C	Monitors of thermal safety and decomposition risks	Stable temperature control without localized hot spots or thermal excursions
Oxygen/gas release	dissolved or vented O_2_	Indicates of potential H_2_O_2_ decomposition	No abnormal oxygen evolution or gas accumulation; gas release should remain within safe venting and process-control limits

## Data Availability

No new data were created or analyzed in this study. Data sharing is not applicable to this article.
